# Reading Speed, Comprehension and Eye Movements While Reading Japanese Novels: Evidence from Untrained Readers and Cases of Speed-Reading Trainees

**DOI:** 10.1371/journal.pone.0036091

**Published:** 2012-05-09

**Authors:** Hiromitsu Miyata, Yasuyo Minagawa-Kawai, Shigeru Watanabe, Toyofumi Sasaki, Kazuhiro Ueda

**Affiliations:** 1 Japan Society for the Promotion of Science, Tokyo, Japan; 2 Graduate School of Human Relations, Keio University, Tokyo, Japan; 3 Department of Psychology, Keio University, Tokyo, Japan; 4 NBS Japan Society of Speed Reading Education, Tokyo, Japan; 5 Interfaculty Initiative in Information Studies, The University of Tokyo/CREST, Japan Science and Technology Agency, Tokyo, Japan; University of Leicester, United Kingdom

## Abstract

**Background:**

A growing body of evidence suggests that meditative training enhances perception and cognition. In Japan, the Park-Sasaki method of speed-reading involves organized visual training while forming both a relaxed and concentrated state of mind, as in meditation. The present study examined relationships between reading speed, sentence comprehension, and eye movements while reading short Japanese novels. In addition to normal untrained readers, three middle-level trainees and one high-level expert on this method were included for the two case studies.

**Methodology/Principal Findings:**

In Study 1, three of 17 participants were middle-level trainees on the speed-reading method. Immediately after reading each story once on a computer monitor, participants answered true or false questions regarding the content of the novel. Eye movements while reading were recorded using an eye-tracking system. Results revealed higher reading speed and lower comprehension scores in the trainees than in the untrained participants. Furthermore, eye-tracking data by untrained participants revealed multiple correlations between reading speed, accuracy and eye-movement measures, with faster readers showing shorter fixation durations and larger saccades in X than slower readers. In Study 2, participants included a high-level expert and 14 untrained students. The expert showed higher reading speed and statistically comparable, although numerically lower, comprehension scores compared with the untrained participants. During test sessions this expert moved her eyes along a nearly straight horizontal line as a first pass, without moving her eyes over the whole sentence display as did the untrained students.

**Conclusions/Significance:**

In addition to revealing correlations between speed, comprehension and eye movements in reading Japanese contemporary novels by untrained readers, we describe cases of speed-reading trainees regarding relationships between these variables. The trainees overall tended to show poor performance influenced by the speed-accuracy trade-off, although this trade-off may be reduced in the case of at least one high-level expert.

## Introduction

The relationship between reading speed and sentence comprehension has been under investigation for more than 50 years (for an overview, see [1]). Poulton [2] discovered that the amount of content retained, which was considered as the criterion for comprehension, increased significantly when the reading speed decreased from around 300 words per minute (wpm) to about 150 wpm. Such reduced comprehension accuracy with increased reading speed has commonly been referred to as the speed-accuracy trade-off [3]–[6]. For example, the speed-accuracy trade-off is reported to occur while skimming text, during which participants somehow grasped gist-relevant information but not the details [7]–[8]. However, evidence for the relationship between reading speed and comprehension is not consistent. A number of studies have shown that reading more quickly improves comprehension [5], [9]. For instance, Walczyk et al. [10] found that encouraging people to read slightly faster than their normal speed by imposing mild time pressure improved sentence comprehension. This improvement was explained in terms of increased motivation and effort resulting from the constraints on reading time (see also [11]). These various empirical findings suggest that the way in which reading speed and comprehension are related can be influenced by multiple factors, including age, motivation, level of reading expertise, and other factors such as instructed reading strategies.

At present, little is known about how patterns of eye movements may be associated with potential relationships between reading speed and comprehension. Also, there is relatively little evidence about the relationships between eye movements, reading speed and comprehension in Japanese. Given such paucity of basic data, it is important to investigate these variables and how they relate to each other in reading Japanese text. The present study was designed to examine how eye movements and speed-accuracy relationships may be interconnected under conditions in which participants read Japanese novels as fast as possible while also comprehending the content, followed by content-related questions. The studies were conducted in a well-controlled setting using a computer-assisted setup customized for different participants to collect basic data from normal adults. An eye-tracking system which provides a natural testing environment without any glasses was used to record eye movements while reading.

Besides collecting such basic data, the present research was also focused on issues of speed-reading. The Park-Sasaki method of speed-reading originated in Korea and was later further developed in Japan [12]–[13]. The method involves organized visual training while forming both a relaxed and concentrated state of mind, as when one is engaged in meditation with the eyes open. According to what the method claims, advanced experts using this method may be capable of reading Japanese sentences faster than 10,000 characters (corresponding to about 4,120 words in English) per minute while following all the characters and lines in order [12]–[13]. However, there has so far been no scientific demonstration regarding comprehension abilities of these experts, which must be evaluated using solid experimental approaches. A growing body of evidence suggests that meditative training based on Oriental Zen practice enhances various abilities including visual perception, attention, and cognition [14]–[17]. Because the Park-Sasaki method essentially involves forming a calm state of mind through an organized series of mental-focusing techniques such as deep breathing with concentration on one’s abdomen and progressive muscle relaxation, this method could be referred to as one variation of meditation-based mental practices that is intended to cultivate capabilities associated with reading. Using Functional Magnetic Resonance Imaging (fMRI), Fujimaki et al. [18] showed that Park-Sasaki experts showed reduced neuronal activation in Broca’s area and Wernicke’s area while speed-reading, suggesting that they may read sentences using fewer phonological processes than untrained participants. Fujimaki et al. [19] found that, in addition to the decrease in neuronal activation in language-related areas, the right intraparietal sulcus showed increased activation during speed-reading, which they suggest may reflect enhanced visuospatial processing in these experts (see also [20]–[21]).

There have been a number of publications on this topic over decades of time, some of which reported seemingly positive effects of speed-reading training [22]–[23]. However, the efficacy of speed-reading methods or the existence of genuine speed-readers remains highly controversial. There are both skepticism about the mechanics of what speed-readers are doing and skepticism about the comprehension of speed-readers [1], [24]. Among works undertaken in English, for example, Just and Carpenter [25] suggested that speed readers can at most extract the gist of the easy, familiar text but not the details. One reason for these critical perspectives may be due to the ways in which English-language writing is organized. That is, in a typical English document, a summary paragraph is provided at the beginning and a topic sentence appears at the beginning of each paragraph, so that readers can catch the main points by skimming. Thus, in this writing system, speed-reading can be accomplished by developing sophisticated skimming strategies [1]. However, this may not be equally true for Japanese writing, because Japanese writing does not usually provide summary paragraphs or topic sentences. This is particularly true for Japanese novels. Thus, scanning or skimming may not be very efficient ways to grasp the main points of Japanese text. The present study is novel in that it quantitatively examined sentence comprehension during speed-reading in Japanese with a unique training method. To appropriately measure the comprehension by these experts, there appear to be several criteria to use in choosing the text: (1) it should be a story that is relatively easy to read and does not require specialized knowledge or schema on any specific area; (2) it should not be widely read; and (3) there should be pieces of comparable length and difficulty for researchers to effectively design multiple sessions. To meet these criteria, it would be best to use a series of recently published Japanese novels and to ask questions on their content following reading.

Accordingly, in addition to investigating relationships between reading speed, comprehension, and eye movement while reading Japanese novels, the present study also examined sentence comprehension skills by speed-reading trainees. As a beginning step regarding the second point, we included three middle-level trainees and one high-level expert within a case-study approach. After reading Japanese novels on a computer screen, participants answered content-related questions, which aimed to examine the extent of comprehension of the novel. Potential differences in eye movements between the trainees and the untrained participants were also examined using an eye-tracking system.

## Materials and Methods

### Study 1

The primary goal of the first study was to obtain basic data on eye movement measures in Japanese adults while reading novels with comprehension. Participants read each of the four stories, around 9,000 characters long, presented on a LCD screen while switching page displays by pressing a response button. They then answered 16 true or false content-related questions. Eye-tracking was done while reading, which enabled examination of the correlations between reading speed and various eye-movement measures including numbers and durations of fixations and saccadic sizes. Given the scores on the comprehension test, it is also possible to examine correlations between levels of comprehension and eye-movement measures. The second purpose was to preliminarily examine the efficacy of this setup to examine abilities and mechanisms associated with speed-reading. In addition to the normal untrained adults, three middle-level trainees of the Park-Sasaki method participated. If these trainees perform well, i.e., show high comprehension scores with short reading time, that would support the efficacy of this testing as well as the reading skills of the Park-Sasaki experts. In contrast, if they fail to perform well, this would either suggest poor performance of the trainees or potential points for improvement associated with this testing. We expected to find that multiple eye-movement measures correlate not only with reading speed but also with comprehension scores, with potential differences between groups.

#### Participants

A total of 17 Japanese adults participated in the reading sessions. Among them, 14 (10 females and 4 males; age, 25–48 years; mean age = 33.9 years, *SD* = 8.2) were university graduate students, research fellows, or company workers with no experience of speed-reading training. The other three participants (female, 59 years old; female, 46 years old; male, 43 years old) were trainees of the Park-Sasaki method of speed reading, who had participated in the training course for 6.2–8.2 years. These three participants were acknowledged to be in the middle level but had not yet attained the high stage of expertise, according to the specific program of the method [13]. The other four (two females and two males; age, 23–30 years; mean age = 26.3 years, *SD* = 2.6) and five (three females and two males; 18–21 years; mean age = 19.4 years, *SD* = 1.0) participants were also included in the two additional control conditions, i.e., no-reading control and gist/detail control, respectively. All participants had normal or corrected-to-normal vision. The present study was approved by the Ethics Committee of Keio University (Approval No. 10013). All participants in both studies gave written informed consent upon agreement cooperate.

#### Settings

The study took place in a dim, sound-attenuated room, where the participants were seated in a comfortable height-adjustable chair. A 58-cm (23.0 inches) TFT LCD monitor (FlexScan EV2333W, EIZO, Ishikawa, Japan) was located on a table in front of the participant. The distance between the monitor and the participant’s eyes was set at 57 cm. One centimeter on the screen corresponded to a visual angle of approximately one degree. The heights of both the monitor and the chair were adjusted for each participant so that he/she could sit in a good posture and gaze at the fixation point in the center of the monitor right in front of him/her. A response device was used that was modified by removing unnecessary keys from a numerical keypad (TK-UFHBK, ELECOM, Osaka, Japan), which the participants put on their thigh. Participants could press a button on the device to switch pages on the display, in a way comparable to a mouse click. These height adjustments and device modifications were made to make the settings similar to that used for speed-reading training, by which the trainees could express their daily abilities. The program for presenting sentence stimuli was written in Microsoft VisualBasic 6.0.

An eye-tracking system (Tobii X120 Eye Tracker, Tobii Technology AB, Danderyd, Sweden) located beneath the monitor in front of the participant recorded the participants’ eye movements on the monitor throughout the reading period. Use of Tobii eye-trackers in studies of reading has become widespread [26], [27], even though it should also be noted that these eye-trackers have less temporal and spatial resolution than other standard eye-trackers. Tobii eye-trackers are also used in studies of other aspects of cognition [28], [29]. The system is non-invasive and is relatively flexible to the head movements of participants, with a measurement accuracy of 0.5 degrees. There is no need to attach additional devices on the participants’ bodies. It is thus regarded as a tool for recording eye movements in a natural situation in which daily cognitive activities are implemented (for more information see [30]). Tobii X-120 is a binocular eye-tracker composed of two infrared cameras. The recording rate was set at 60 Hz. Tobii Studio software (ver. 1.7.3) was used to set up, execute, and later analyze the test sessions.

#### Materials

**Figure 1:**
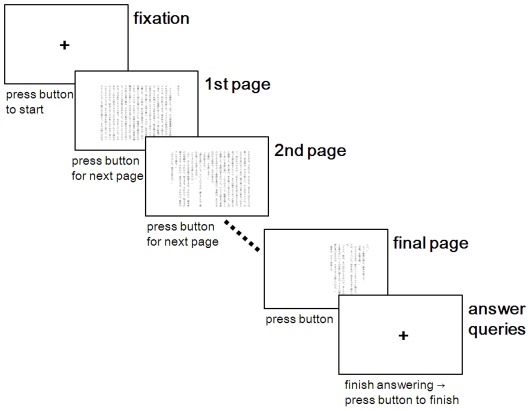
Diagram of a test session in Studies 1 and 2. Japanese sentences shown here, i.e., part of the well-known novel *Hashire Melos* [Run, Melos!], are the samples used for the instruction. At the beginning fixation stage, participants were allowed to take sufficient time before starting each test session.

The texts used for the test sessions were four stories selected from the same series of Japanese novels (*99-no-namida* [99 tears] by Lindapublishers Co., Ltd., Tokyo, Japan) published between the years 2009 and 2010: *Tonari no hatsumeika* (Inventor next door) [31]; *Samui yoru no pizza* (Pizza on a cold night) [32]; *Ryu to issho* (Together with Ryu) [33]; and *On-okuri* (Pay it forward) [34]. These stories are referred to hereinafter as Story 1, 2, 3, and 4, respectively. These four stories were 8,046–9,617 characters (5,029–6,011 Japanese words) long (9,004 characters/5,628 Japanese words on average). All four novels involved relatively easy content on daily events of general people in the present day. All participants declared that they had not read any of these stories prior to the study. As is typical in Japanese text, the sentences in these novels used a combination of three writing systems, i.e., *kanji* (morphogram), *hiragana* (syllabogram), and *katakana* (syllabogram). The sentences were arranged in a vertical direction from up to down and subsequently from right to left, as is the usual style of text arrangement in Japanese novels. Japanese words consist of one or multiple characters (mean: about 1.6 characters), usually classified into ten parts of speech. However, unlike the English writing system, no spaces were inserted between words, and gaps between words are less apparent than in English. Consequently, word-based counting is less common than character-based counting in Japanese writing. Sentences were presented at the center of the monitor within an area 20 cm wide and 14 cm high. Each page display contained 392 characters (about 245 words) on average, in 20 lines (see **Figure 1** for examples of the page displays). Each line included 25 characters (about 16 words), i.e., 1.8 characters per degree of visual angle. Each story corresponded to 21–25 pages on the monitor. For the instruction, the text of another well-known novel, *Hashire Melos* (Run, Melos!) by Osamu Dazai (9,795 characters/6,122 words long), was used.

#### Procedure

Instruction on the general procedure using *Hashire Melos* (Run, Melos!) as an example was followed by the test sessions for both the trainees and the untrained participants. The flow of each session is depicted in **Figure 1**. Prior to each test session, regular full-screen calibration check of the eye-tracker was performed. During calibration, a target circle moved in animation on the display, which participants were required to follow with their eyes until the target made brief stops at nine different points. Following calibration, differences between the actual locations of these stop points and the gaze points calculated by the eye-tracker were displayed using lines. Calibration was deemed acceptable if these lines remained within the area indicated for each stop point. Mean measurement accuracy of 0.5 degrees was regarded to be achieved with these criteria. Otherwise, if the longer lines indicated larger errors or if the software displayed that there were not enough calibration data, recalibration was performed until acceptable calibration was obtained. After a fixation point appeared at the center of the display, participants were allowed to start reading in their own good time. Pressing a response button once displayed the first page of the story. After reading through each page once, participants pressed the button once and the display switched to the next page. Participants were instructed to read sentences as fast as possible while understanding the content as well. All participants were also told to maintain both a good posture and a calm, relaxed state of mind throughout the reading period, so that trainees could exhibit their reading capabilities by performing in the same ways as they usually do during the training. In addition, they were instructed not to move their head or speak, to reduce artifacts in the eye-tracking data, unless they wanted to terminate the session. Immediately after reading through the final page, another press of the button resulted in the reappearance of the fixation cross, and participants were required to answer 16 true or false questions presented on a sheet of paper set in front of them on the table. The sheet, one for each session, was set face-down before each session through the reading period, after which participants turned it over to fill in the answers with a pen. The questions concerned the plot of the novels, and were designed so that they would be relatively easy to answer if the reader comprehended the content of the story but not if they read without appropriate understanding of the content. Examples of the questions were in the form of sentences such as: *Q. The boy thought that the old patient is playing many tricks in an attempt to ease his terror of death* (Story 1). *Q. The heroine realized that her dead mother, whom she once hated, actually loved her a lot* (Story 4). Participants gave true or false marks to each sentence based on whether it matched the plot of the story. Immediately after finishing the answers, participants pressed the button once more to terminate the session. Between the test sessions, participants were allowed to take a rest period if they wished.

Participants included in the additional two control conditions, i.e., no-reading control and gist/detail control, were non-speed-readers and were ignorant of the purpose of the study. No-reading control participants were simply presented with the question sheets and filled in the answers to the questions for all the test stories used in this study without reading the novels. Gist/detail control participants read the text, questions, referred to the correct answers to the questions and rated each question on a scale of one to ten, based on the extent to which the question reflected the (1) gist and (2) detail of the stories.

#### Eye-tracking data analysis

Eye-movement data post-processed by Tobii Studio software showing timestamps in milliseconds and coordinates of each fixation were used for the subsequent analyses. A fixation was scored if the gaze remained stationary within a radius of 35 pixels (about 0.9 degrees in visual angle); otherwise, the recorded sample was defined as part of a saccade. The following variables were used to indicate characteristics of eye movements: numbers of leftward and rightward saccades in the X axis; numbers of downward and upward saccades in the Y axis; mean size of saccades (absolute value integrating X and Y components) and its X and Y components separately with their variances; size of saccades in X and Y shown separately for the first-pass reading; mean duration of fixation and its variance; and first-pass reading time (i.e., sum of all fixation times during the first pass) and look-back time within each session. First-pass in this context was determined as all the fixations made from first entering each line of the text until leaving it. First-pass measures should help to consider to what extent comprehension was made during first-pass, or during re-reading to understand further details of the text. Saccades larger than 200 pixels (about 5.3 degrees in visual angle) in the rightward direction for the X axis or in the upper direction for the Y axis were analyzed separately, because these were regarded as saccades made to proceed to the beginning of the next page display and to the beginning of the next line (i.e., “return sweeps”), respectively. Regressive saccades were defined as those moving in the rightward direction for the X axis, or in the upward direction for the Y axis within each line. Correlations were examined for untrained participants between these measures and three independent variables: reading speed (characters read per minute), proportions of correct answers to the questions (comprehension scores), and residual values in Y from the regression line in the speed-accuracy plot, which was assumed to reflect the excellence of performance in the relationships between reading speed and comprehension scores.

### Study 2

Whereas the first study revealed correlations between reading speed, accuracy, and eye movement, it also revealed poor performance in the trainees. Potential problems and points for improvement are: (1) participants should be those at the high stage of expertise, who can both comprehend and remember the content while reading at a high speed; and (2) the present instruction to read as fast as possible while also comprehending may have been misleading, because it could have urged participants to accelerate reading speed while sacrificing comprehension. It would be better to more strongly encourage participants to focus on comprehension. Thus, in the second study, we included a high-level expert as well as new untrained participants and modified the instructions so that participants would prioritize comprehension. We expected that the expert would show performance exceeding that of the control participants, while potentially showing different eye-movement patterns as well.

#### Participants

An advanced expert of the Park-Sasaki method (FT; female, 59 years old) participated. Prior to participation FT had participated in the training course for 7.4 years and attained the high stage of expertise. According to the specific program of the method, she was reportedly capable of reading more than 120,000 characters per minute using the familiar sentences in the training textbook of the method [13]. FT reported that her daily reading speed in private when reading Japanese books for pleasure was between 5,000 and 8,000 characters per minute. That is, she reported that the reading speed which she can practically show for unfamiliar text was much lower than when reading familiar text during training. The other 14 Japanese university undergraduate students having no experience with speed-reading training (10 females and 4 males; age, 18–19 years; mean age, 18.9 years, *SD* = 0.3) participated as the untrained control group. For the no-reading control and gist/detail control conditions, data from the same participants as in Study 1 were used, except that data for one additional story used in Study 2 were included. In addition, the other four participants (3 females and one male; 19–39 years; mean age, 24.0 years, *SD* = 8.7) had another control condition referred to as FT’s-speed control. All participants had normal or corrected-to-normal vision.

#### Settings, materials, and procedure

The same setup as in Study 1 was used for Study 2. The materials were the same as in Study 1 except that one additional story from the same series of novels was used for the test, which now involved five sessions in total. This new story, *Issho-no-onegai* (I’ll never ask for anything again) [35], was 9,046 characters (5,654 Japanese words) long and corresponded to 23 pages on the display. All participants declared that they had not previously read any of the stories used for the test. The procedure was the same as in Study 1 except that five test sessions were implemented and that the instructions were modified. That is, participants were told to read the sentences while comprehending the content, so that they would be able to answer content-related questions presented following reading. While maintaining that level of understanding, they were instructed to read not slowly nor lazily but as rapidly as possible. FT’s-speed control participants were non-speed-readers and participated in all the five test sessions following FT’s participation. These participants answered the questions after having each page display switched automatically after the same presentation time as FT had. They were instructed that the pages would be switched automatically at speed and that they should do their best to comprehend the stories without pressing response buttons by themselves.

#### Eye-tracking data analysis

Eye movement measures by the eye-tracker were analyzed and examined generally in the same manner as in Study 1. Examination of correlations between eye-movement measures, reading speed and comprehension scores, already reported in Study 1, were not duplicated in this study. In order to identify the parts of the sentence display where the participants’ gaze was frequently fixated, the sentence area of 20×14 cm was divided into subareas of every 2 cm for both the vertical and horizontal axes. These subareas are referred to as X1 to X10 (from left to right) for the horizontal axis and as Y1 to Y7 (from top to bottom) for the vertical axis. Durations of fixations at each of these subareas were examined. All fixations, including those during first-pass reading, re-reading, and return sweeps, were included in this analysis.

## Results

### Study 1

#### Reading speed and comprehension scores

**Figure 2:**
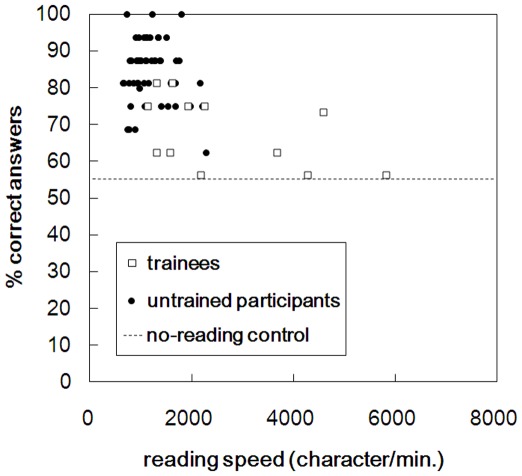
Results of Study 1: reading speed in the characters read per minute and comprehension scores, percentage of correct answers to the questions, by the 14 normal participants (“untrained participants”) and three trainees of the speed-reading method (“trainees”). Each dot shows data obtained from each reading session. The dashed line indicates the averaged comprehension score by no-reading control participants who gave answers to questions without reading the novels.

Data from all the test sessions were included in the analysis, but one untrained participant (female, 30 years old) quit after finishing two sessions. The average score of the no-reading control participants across the four stories was 55.5%, which was considered to reflect the baseline “zero-comprehension”. The mean rating values regarding the questions by the gist/detail control participants were 5.3 for gist and 7.4 for detail, showing that the questions reflected both the gist and the detail of the stories. Reading speed and percentages of correct responses to the questions for both the trainees and the untrained participants are shown in **Figure 2** and in **Table 1**. Data for each test session is plotted in **Figure 2**. When both the untrained participants and the trainees were involved, reading speed and proportions of correct answers to the questions revealed a significantly negative correlation (*R* ^2^ [N = 66] = −0.528, *p*<0.001). A comparable negative correlation was found when each group was separated, though these did not reach significance (untrained participants: *R* ^2^ [N = 54] = −0.146, *p* = 0.293; trainees: *R* ^2^ [N = 12] = −0.500, *p* = 0.098). In addition, Mann-Whitney’s *U* tests showed that comprehension scores for both groups were significantly higher than those of the no-reading control group (untrained participants: *U* (N = 54, 16) = 16.500, *p*<0.001; trainees: *U* (N = 12, 16) = 38.500, *p* = 0.007). Mean reading speed was numerically 2.1 times faster, and mean proportion of correct answers was numerically 15.7% lower, in the trainees than in the untrained participants (**Table 1**). For both reading speed and comprehension score measures, we conducted Mann-Whitney’s *U* tests in order to make comparisons between the trainees and the untrained participants. Data for all sessions were used for this analysis. Reading speed turned out to be significantly faster in the trainees than in the untrained participants (*U* (N = 54, 16) = 105.000, *p*<0.001). Comprehension scores, however, were significantly lower in the trainees than in the untrained participants (*U* (N = 54, 16) = 67.500, *p*<0.001). These data show that, whereas the trainees tended to read faster than the untrained participants, their accuracy on comprehension scores was lower, though higher than that of the no-reading control participants. In addition, Mann-Whitney’s *U* tests compared comprehension scores between questions with above- and below-average scores of gist/detail rating values, for both the untrained participants and the trainees. No significant differences in comprehension scores were found between higher and lower gist (untrained participants: *U* (N =  34, 30) = 481.500, *p* = 0.694; trainees: *U* (N =  34, 30) = 443.500, *p* = 0.343) or detail (untrained participants: *U* (N =  25, 39) = 428.500, *p* = 0.405; trainees: *U* (N =  25, 39) = 392.500, *p* = 0.166) rating values. Thus, it was not shown that the extent to which the questions reflected the gist/detail of the stories influenced the comprehension scores.

**Table 1:** Summary of reading speed, comprehension scores, and eye-movement measures for both the untrained participants and the trainees in Studies 1 and 2.

speed, accuracy, and eye-movementmeasures	Study 1		Study 2	
	untrained participants	trainees	untrained participants	trained individual(FT)
	mean (variance)	mean (variance)	mean (variance)	mean (variance)
reading speed (character/min)	1236.6	2600.2	1193.4	5644.0
% accuracy on questions	83.8	68.1	84.9	77.5
N leftward saccades	870.9	602.1	924.7	258.0
N rightward saccades	575.3	384.9	635.3	49.0
N downward saccades	1184.8	643.8	1278.1	220.6
N upward saccades	261.4	343.2	281.9	86.4
N return sweeps	294.8	205.3	297.5	60.2
saccade size: total (degree)	3.6 (7.2)	3.7 (4.8)	3.6 (7.9)	3.0 (2.9)
saccade size in X: total (degree)	0.3 (0.7)	0.4 (1.4)	0.2 (0.7)	1.1 (1.3)
saccade size in Y: total (degree)	1.7 (5.1)	1.2 (9.5)	1.6 (5.3)	0.7 (4.8)
saccade size in X: first pass (degree)	0.3 (0.7)	0.5 (1.4)	0.3 (0.7)	1.1 (1.3)
saccade size in Y: first pass (degree)	1.8 (4.7)	1.4 (9.0)	1.8 (5.0)	0.7 (4.8)
fixation time (msec)	268 (15525)	224 (8876)	278 (24098)	274 (10527)
first-pass reading time (sec)	441.2	240.0	498.0	99.3
look-back time (sec)	26.4	22.8	27.3	0.1

Saccade sizes in X and Y are shown both for all saccades and for saccades during the first pass. Saccade sizes are presented in terms of degrees of visual angle. Depicted are the representative values that were averaged across sessions, and then across participants. For saccade size and fixation time measures, variances are also shown in parenthesis.

#### Eye-movement measures and correlations

All analyzed eye-movement measures together with reading speed and comprehension score measures for the trainees and the untrained participants from both studies are summarized in **Table 1**. The three trainees showed numerically smaller fixation times, larger saccade sizes in X and smaller saccade sizes in Y, and fewer return sweeps, although these numerical differences were not very distinctive. In addition, results of correlation analyses between the independent variables, i.e., reading speed, comprehension scores, and residual value from linear regression in the speed-accuracy plot, and the eye-movement measures for the 14 untrained participants are summarized in **Table 2**, to show relationships between these measures for normal readers.

**Table 2:** Results of Study 1.

eye-movementmeasure		reading speed		% correct answers		residual value in y	
		*R^2^ (N = 54)*	*p*	*R^2^ (N = 54)*	*p*	*R^2^ (N = 54)*	*p*
saccade size: total (pixels)	mean	0.124	0.185	0.036	0.398	0.071	0.306
	variance	−0.468	<0.001***	0.046	0.369	−0.084	0.273
saccade size in X: total (pixels)	mean	0.897	<0.001***	−0.190	0.085	0.060	0.333
	variance	0.362	0.004**	0.245	0.037*	0.346	0.005**
saccade size in Y: total (pixels)	mean	−0.175	0.103	−0.038	0.394	−0.086	0.267
	variance	0.505	<0.001***	0.084	0.272	0.226	0.051
saccade size in X: first pass (pixels)	mean	0.893	<0.001***	−0.186	0.089	0.063	0.325
	variance	0.355	0.004**	0.247	0.036*	0.347	0.005**
saccade size in Y: first pass (pixels)	mean	−0.168	0.112	−0.005	0.486	−0.052	0.355
	variance	0.541	<0.001***	0.089	0.260	0.241	0.040*
fixation time (msec)	mean	−0.577	<0.001***	−0.219	0.056	−0.381	0.002**
	variance	−0.571	<0.001***	−0.113	0.207	−0.273	0.023*
% rightward regression		−0.234	0.044*	0.260	0.029*	0.195	0.079
% upward regression		0.454	<0.001***	0.058	0.338	0.185	0.090

Shown is the summary of correlations between independent variables (reading speed, comprehension scores, and residual values in the speed-accuracy linear regression) and eye-movement measures (saccade size, fixation time, and proportion of regression) across all the sessions from untrained participants. *R^2^*: Pearson correlation coefficient.

* : *p*<0.05;

** : *p*<0.01;

*** : *p*<0.001.

##### Reading speed

Correlations between reading speed and eye-movement measures for untrained participants revealed a number of significant tendencies. First, faster reading corresponded to shorter duration of each fixation and smaller variance in fixation time. Second, faster reading corresponded to smaller variances in total saccade sizes. Analyzed separately for X and Y components, faster reading corresponded to larger saccade size in X (in the leftward direction), but not in Y (in the downward direction). Variances in saccade sizes both in X and Y were larger in fast readers than in slow readers. Comparable trends were found for saccade sizes in X and Y during the first pass. Finally, faster reading corresponded to smaller proportions of rightward regressive saccades, i.e., moving rightwards back to the sentences where the participant had already read, but to larger proportions of upward regressive saccades within each line.

##### Comprehension scores

Correlations between comprehension scores and eye-movement measures generally failed to reveal strong statistical significance, compared with the former analyses regarding reading speed. One statistical significance was found for the higher comprehension scores corresponding to larger proportions of rightward regressive saccades, as opposed to the case for reading speed.Variances in saccade sizes in X were larger in readers with higher comprehension scores than in those with lower scores, both for all saccades and saccades during the first pass. Otherwise no statistically significant correlations were revealed.

##### Residual value from linear regression in the speed-accuracy plot

Mean residual value was 2.30 for the untrained participants and −5.86 for the trainees. These numerical data show lower performance by the trainees than by the untrained participants within the speed-accuracy plot. For the untrained participants, shorter duration of each fixation and smaller variance in fixation time corresponded to larger residual values. Also, larger variances in saccade sizes both in X and Y corresponded to larger residual values, more apparently for saccades during the first pass than for all saccades. These show that untrained readers with higher performance within the speed-accuracy plot tended to make fixations with shorter and less variable duration, and to make saccades more variable in size for both the vertical and horizontal axes, compared with those with lower performance. The other correlations involving total saccade sizes and regressive saccades failed to reach statistical significance.

### Study 2

#### Reading speed and comprehension scores

**Figure 3:**
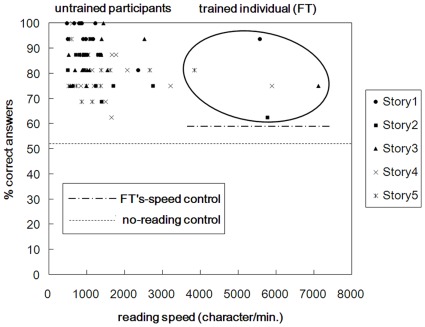
Results of Study 2: reading speed in the characters read per minute and comprehension scores, i.e., percentage of correct answers to the questions, by the untrained participants and the trained individual (FT). Data for FT are enclosed by an ellipse. Each dot shows data obtained from each reading session. The dashed line indicates the averaged comprehension score by no-reading control participants, who gave answers to questions without reading the novels. The long dashed dotted line indicates the averaged comprehension score by FT’s-speed control participants, who answered questions after having page displays switched in the same presentation times as FT.

Data from all the test sessions were included in the analysis, but one untrained participant (male, 19 years old) quit after completing four test sessions other than Story 2. The average scores of the participants from no-reading control and FT’s-speed control groups across the five stories were 52.2% and 58.9%, respectively. The average rating values regarding the questions by the gist/detail control participants were 5.0 for gist and 7.1 for detail, showing that the questions reflected both the gist and the detail of the stories. Mean reading speed was numerically 4.7 times faster and mean proportion of correct answers to the questions was numerically 7.4% lower, in the trained individual (FT) than in the untrained participants. **Figure 3** depicts reading speed and proportions of correct answers in both FT and the untrained participants. Each test session is plotted separately using different markers for different stories. Mann-Whitney’s *U* tests showed that comprehension scores for both untrained participants and FT were significantly higher than those of the no-reading control group (untrained: *U* (N = 69, 19) = 21.500, *p*<0.001; FT: *U* (N = 5, 19) = 6.000, *p* = 0.003). In addition, comprehension scores for FT were significantly higher than those of the FT’s-speed control group (Mann-Whitney’s *U* test; *U* (N = 5, 20) = 10.000, *p* = 0.004).

For both reading speed and comprehension score measures, we conducted Mann-Whitney’s *U* tests to make comparisons between FT and the untrained participants. Reading speed was significantly faster in FT than in the untrained (*U* (N = 5, 69) < 0.001, *p*<0.001). Comprehension scores, however, did not significantly differ between FT and the untrained participants (*U* (N = 5, 69) = 104.500, *p* = 0.135). Thus, whereas the reading speed of the trained individual (FT) was significantly faster than that of the untrained participants, the accuracy of the comprehension test for FT was statistically as high as that for the untrained. Comprehension scores between questions with higher and lower gist/detail rating values were compared, comparable to Study 1. No significant differences were found between higher and lower gist (untrained: *U* (N =  42, 38) = 688.000, *p* = 0.275; FT: *U* (N =  42, 38) = 776.000, *p* = 0.769) or detail (untrained: *U* (N =  38, 42) = 650.000, *p* = 0.142; FT: *U* (N =  38, 42) = 696.000, *p* = 0.174) rating values. Thus, there was no evidence that the extent to which the questions concerned the gist or the detail may have influenced comprehension.

#### Eye-movement measures

**Table 1** summarizes eye-movement measures for the trained individual (FT) and the untrained participants respectively. Data obtained for the untrained participants in Study 2 were generally similar to those obtained for the untrained participants in Study 1, despite the modified instructions. Specifically, numerical comparisons between the untrained participants and FT showed the following trends. Fixation time was not largely different between these participants, but its variance was smaller in FT than in the untrained participants. Total saccade size (absolute value integrating X and Y components) and its variance were smaller in FT than in the untrained participants. The X component of the saccade was larger, and the Y component was smaller, in FT than in the untrained participants. The variance of the X component was larger, and the variance of the Y component was smaller, in FT than in the untrained participants. Comparable trends were observed for saccades during the first pass. Most saccades for FT were made during the first pass, with the mean look-back time as short as 0.1 s. In addition, the number of return sweeps and the proportion of rightward regressive saccades were smaller, although upward saccades were more frequent, in FT than in the untrained participants.

**Figure 4:**
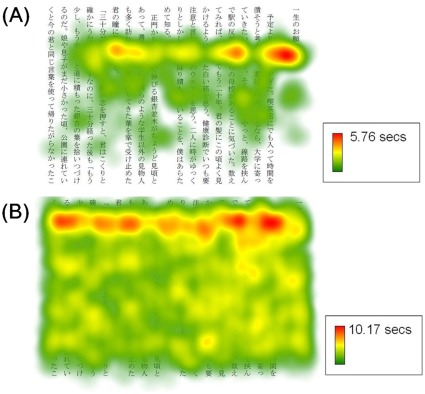
Heat maps showing typical distributions of fixation times in Study 2. Fixation times for each area of the sentence display are shown in different colors. Each figure shows data for the representative test session using Story 1, (**A**): trained individual (FT) and (**B**): an untrained participant (female, 19 years). The figures were drawn using the Tobii Studio software (ver. 1.7.3).

**Figure 5:**
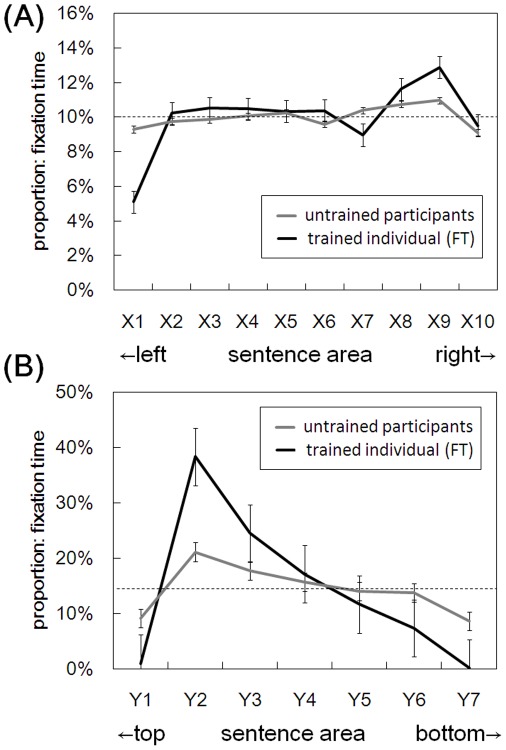
Proportion of fixation times at each of the sentence areas for the trained individual (FT) and the untrained participants in Study 2. Shown are the data averaged across stories and then across participants for the untrained. The sentence area was divided into subareas of every 2 cm for the horizontal (X1–X10; [A]) and vertical (Y1–Y7; [B]) axes, respectively. Dashed lines show the proportions expected by the chance level (i.e., 1/10 for [A] and 1/7 for [B]). Error bars indicate standard errors of the mean.

To visualize the differences in looking patterns between the trained in individual (FT) and the untrained participants, **Figure 4** depicts heat maps showing durations of fixations at each part of the sentence display from typical sessions for FT (**A**) and the untrained (**B**) participants. Further, **Figure 5** illustrates proportions of fixation times at each of the horizontally (**A**) and vertically (**B**) divided subareas of the sentence display. These divisions are referred to as subareas X1–X10 and Y1–Y7, respectively. Mann-Whitney’s *U* tests for each subarea made comparisons between FT and the untrained participants. A false discovery rate (FDR) method [36] was used to correct multiple comparisons. Regarding horizontal divisions (**Figure 5**(**A**)), for X9 the proportion was significantly higher (*U* (N = 5, 60) = 23.000, *p*<0.05, corrected), and for X1 the proportion was marginally lower (*U* (N = 5, 60) = 47.000, *p*<0.10, corrected), in FT than in the untrained participants. No significant differences were found for the other subareas (X2–X8; X10) (*U* (N = 5, 60) = 60.000–146.000, *p*>0.10, corrected). Regarding vertical divisions (**Figure 5**(**B**)), for Y2 and Y3 the proportions were significantly higher (*U* (N = 5, 60) = 2.000–34.000, *p*<0.05, corrected), and for Y4 marginally higher (*U* (N = 5, 60) = 61.000, *p*<0.10, corrected), in FT than in the untrained participants. On the other hand, for all the remaining subareas (Y1, Y5, Y6, and Y7), the proportions were significantly lower in FT than in the untrained participants (*U* (N = 5, 60) = 3.000–67.000, *p*<0.05, corrected). Thus, the trained individual (FT) looked at the relatively upper part of the sentence area more frequently, but the lower part less frequently, than the untrained participants. Also, whereas FT frequently looked near the right end of the sentence area, she tended to look near the left end less frequently than the untrained participants.

## Discussion

### Study 1

The results revealed various relationships between reading speed, comprehension scores, and eye-movement measures in untrained participants and the trainees. In particular, eye-tracking data for untrained participants revealed multiple significant correlations between reading speed and comprehension scores and eye-movement measures. Specifically, faster reading was associated not only with shorter fixation times and fewer/more rightward/upward regressive saccades, but also with larger horizontal saccadic movements. Saccade sizes during the first pass generally produced trends similar to those for all saccades. This suggests that eye-movement strategies, such as larger saccade sizes in X in fast readers, largely reflect those during first-pass reading of lines of text. In addition, larger variances in saccade sizes in faster reading suggest the use of irregular or unsteady eye-movement strategies as reading rate accelerated. On the other hand, comprehension scores generally revealed less apparent correlations with the eye-movement measures, compared with reading speed. However, higher scores on the comprehension test were associated with more rightward regressive saccades. This could be interpreted as moving back to the previous lines in order to obtain more accurate understanding of the content. Finally, residual value from linear regression in the speed-accuracy plot showed that shorter fixation times with smaller variances and larger variances in saccade sizes corresponded to larger residual values. These support the notion that relatively excellent reading performance was achieved by using more variable eye-movement strategies. These correlations were found in a situation in which untrained participants read as fast as possible while also comprehending the content, that is, while performing at their best. Thus, it seems possible that these data may reflect patterns generally observed when normal native participants read comparable Japanese text, while using reading strategies to read faster than their usual daily speed.

With regard to speed-reading, on the other hand, the results revealed rather poor performance in the Park-Sasaki trainees. That is, the negative speed-accuracy correlation involving both untrained participants and the trainees suggested a speed-accuracy trade-off. This trade-off appears consistent with previous studies: even though a small increase in reading speed is not known to impair comprehension [10], a reading rate that is further increased may negatively impact comprehension [5]. Whereas the trainees in this study read faster overall than the untrained participants, their comprehension scores were lower than the untrained. This suggests that speed-reading can lead to poor comprehension at a certain level of expertise. However, the trainees’ comprehension scores were at least higher than those of the no-reading control participants. Thus, instead of gazing over the sentences with no understanding, at least some form of comprehension might have been made using the trainees’ reading strategies. Appropriate procedural modification may improve outcomes in terms of drawing out the abilities potentially possessed by the trainees. The second study addressed these issues by including a high-level expert and modifying the instructions.

### Study 2

The reading speed of the trained individual (FT) was numerically 4.7 times that of the untrained participants, and her comprehension scores were significantly higher than the zero-comprehension level for all the five test sessions. This was statistically no different from the comprehension scores of the untrained participants, albeit numerically 7.4% lower on average. FT’s comprehension scores were also higher than those of the untrained participants having comparable exposure to the text (FT’s-speed control). This suggests that untrained participants might not produce results comparable to FT by simply adopting different strategies to read as fast as FT. After finishing all the sessions, FT reported that she understood the stories as a whole and gave details about what types of questions she found difficult to answer; for example, those on the specific characteristics of a person in one story. Thus, with reading speed much faster than those of all the untrained participants the trained individual (FT) maintained levels of sentence comprehension close to those of untrained participants, while the questions reflected both the gist and the detail of the stories. FT’s performance thus appears to show reduced speed-accuracy trade-off as observed in Study 1.

Analyses of eye movements during these sessions revealed that fixation time and saccade sizes of FT were numerically not distinctively different from those of the untrained participants. However, FT tended to make saccades larger in horizontal, leftward directions rather than in vertical, downward directions typical of the untrained participants. In addition, rightward regressive saccades and return sweeps were less frequent in FT compared with the untrained participants. These fewer return sweeps, larger saccade sizes in X than one degree, and more frequent upward saccades in FT than in the untrained participants, which appears consistent with but more apparent than in the trainees of Study 1 (**Table 1**), suggest that she made horizontal saccades in the upper-left (/lower-left) direction to cross lines forward. Further, distributions of fixation coordinates also suggested that FT mainly made horizontal eye movements and directed her pupils on limited parts of the sentence display (**Figure 4**). Specifically, fixation of FT concentrated on around 2–4 cm from the top of the sentence area. Whereas FT frequently looked near the right end of the area, near the left end there were some subareas she rarely looked at. First-pass analyses suggest that these looking patterns by FT were almost always made during the first pass and not during re-reading. These tendencies were in contrast to those of the untrained readers, who typically read in line-by-line ways and thus gazed over almost all parts of the sentence area, sometimes re-reading the same lines after the first pass. These participants also tended to frequently look near the right end and near the top of the sentence display, seemingly in order to determine the beginnings of each page and each line. However, these tendencies seem less apparent compared with those of FT (**Figure 5**).

Given the comprehension scores, it appears that even though FT did not direct her gaze over all characters or lines while speed-reading, she appeared to understand the content of the stories to a certain extent. It might not be, for example, that FT obtained information from only a few words on which her eyes were fixated during the first pass: such strategies might not have produced her comprehension scores, as the lower scores by FT’s-speed control participants suggest. The Park-Sasaki method teaches looking strategies in which the trainee shifts one’s attention ahead while never feeling the movement of one’s own eyes. Consequently, the trainee learns to move the eyes horizontally while seemingly viewing all the characters in order. The present results thus may support the possibility that at least one expert might have developed such looking strategies through training to a level at which she can grasp the content of sentences to a certain extent.

### General Discussion

The present study examined relationships between reading speed, scores on comprehension tests and eye-movement measures while native participants read Japanese contemporary novels. In addition to normal untrained participants, four trainees of the Park-Sasaki speed-reading method were included in the two case studies. In Study 1, the Park-Sasaki trainees showed poor sentence comprehension performance influenced by the speed-accuracy trade-off. In addition, for normal untrained readers, multiple statistically significant correlations were revealed between reading speed, comprehension scores, residual value from linear regression in the speed-accuracy plot, and eye movement measures. In Study 2, one trained speed reader (FT) showed comprehension scores approaching those of the untrained participants while reading faster. This single case suggested the possibility of reduced speed-accuracy trade-off. FT’s eye-movements appeared different from those of the untrained participants in that she mainly made saccades in horizontal directions while looking at limited parts of the sentence display with little re-reading, scarcely moving her eyes in the vertical direction as did the untrained readers.

Some of the the conclusions from the present studies can be discussed in relation to previous work in English. The speed-accuracy trade-off as found in Study 1 is consistent with other previous works [7]–[8]. It seems worth noting that, unless the trainees were in the high-level of expertise in the Park-Sasaki method, speed-reading can lead to rather poor levels of comprehension. Actually, as indicated in **Figure 2**, when the trainees read at speeds similar to untrained participants, their comprehension scores were no better, if anything poorer, than untrained participants. When the trainees read at higher speeds their comprehension performance often appeared to be little different from that of participants who did not even read the texts, i.e., no-reading control. Thus, even the gist of the text sometimes may not have been comprehended by the trainees. As Sasaki [12] states, the Park-Sasaki method is based on reading all the characters and lines in order, depending neither on skimming nor scanning. Participants in Study 1 were the middle-level trainees who had mastered the specific strategy of looking at characters in order and at a high speed but who had not yet attained the more advanced stage of the method, so that the looking strategy can be used while sufficiently comprehending the content. This difficulty was true for the present comprehension tests that required not only understanding of what they are reading at each given moment but also the ability to store the content in short-term memory until the whole story was finished [37]. Thus, answering the questions after reading through the sentences once may have been demanding for the trainees. Even though it seems that the trainees could move through the text more quickly than the untrained participants, the data suggests that these trainees failed to achieve the same levels of comprehension that they might achieve by reading in a standard manner. These poor performances need to be emphasized, considering that they have participated in the training for a number of years.

Regarding Study 2, numerically similar comprehension scores between the trained individual (FT) the and untrained participants in Study 2 might be somewhat better than Just and Carpenter [25] reporting that speed-readers in English are capable of at most grasping the gist of the easy, familiar text but not the details. That is, we used novel text and the questions reflecting both gist and the detail of the stories, based on the rating scores by the gist/detail control participants. Also, the extent with which each question reflected gist/detail did not seem to have influenced comprehension scores. However, FT’s comprehension scores were numerically lower than those of the untrained participants. It seems possible that FT’s scores would have been improved, perhaps exceeding those of the untrained participants, if she had read at a standard speed. To what extent this improvement actually occurs seems to require further examination.

There appear to remain a series of further issues that need to be carefully considered or addressed in the future, before making better interpretation of the data and conclusive remarks regarding efficacy of the Park-Sasaki method. First, small sample sizes in the present study clearly impose limitations on the conclusions drawn about speed-reading. That is, conclusions related to speed-reading based on the present study require particularly cautious considerations given that they mainly derive from numerical differences or non-parametric tests. Even though the speed-accuracy trader-off appeared to be reduced for the trained individual in Study 2, this single case could be insufficient for researchers to conclude that the trade-off is overcome in high-level or top-level Park-Sasaki experts. More trained participants who did not participate need to be included in the future when they have achieved sufficiently high levels of expertise, so that the present results could be generalized to a larger population. There were also age differences between the trainees and the untrained participants, in particular in Study 2. It would thus be more ideal in the future to control the ages of these participants between groups.

Second, what types of looking and reading strategies the Park-Sasaki trainees used has to be cautiously considered. The pattern of eye movements shown by the trained individual in Study 2 (**Figure 4**(**A**)) indicates that most fixations occurred within a small area of the text. Nevertheless, comprehension scores by this participant indicate that a certain level of comprehension was made in the absence of fixations over a broader area. One possibility could be that the expert has developed sophisticated quick-reading strategies such as skimming or scanning [1], [25]. However, as the lower scores from the FT’s-speed control participants suggest, the trained individual might not produce comparable scores by, for example, sampling a small proportion of the words and using heuristics to guess the plot (although this surely requires further rigorous experimental examination). In addition, the questions concerned both the gist and the detail of the stories. The extent to which the question reflected the gist/detail did not seem to have influenced comprehension. The possibility that the perceptual span may be extended in the trainees has to be further addressed either by using a moving window method or by using modified tasks that allow for analysis of comprehension for details of the text where participants’ eyes were not fixated. However, it could nevertheless be possible that highly skilled looking and reading mechanisms unique to this method might be associated with extended perceptual span. For example, Rayner [38] used a moving window technique and showed that perceptual span was larger in skilled readers than in beginning or less proficient readers. It might also be possible that multiple words can be processed in parallel under certain situations with short sentence presentation times, in particular when extracting the gist of the text [39].

Third, a major problem in comparing the trainees and the untrained participants is that there may well be underlying differences between these participants that are not simply associated with the training procedure: (1) Those who self-selected themselves for training may be generally more highly motivated to read efficiently. (2) Those who are successful in the training process and hence choose to continue may have individual differences that make them more suited to this training method. For example, they may have particularly good parafoveal vision or they may be particularly skilled readers from before training, so that they would be better able to accurately identify words further from the fixation point that are visually degraded. (3) Those who select themselves for training may simply benefit from having more reading practice. To clarify these issues, further to comparing advanced experts and untrained participants, it would be required to randomly assign naïve participants to training and no-training groups, and make comparisons between pre- and post-training. Also, another potential approach could be to employ within-participants designs with the same readers taking either a speed-reading or normal-reading strategies, hence controlling for other variables including reading practice, ability to use heuristics, age, and so on.

Fourth, the nature of comprehension being assessed in this study needs to be carefully considered. In the present task, the comprehension questions asked about the plot of the stories. This appears appropriate when examining comprehension of the context in reading short contemporary novels. Ratings on the questions by gist/detail control participants suggested that they reflected both the gist and the detail of the stories. The extent to which the questions reflected the gist/detail of the stories did not seem to have effect on comprehension. However, it should be noted that the degree of comprehension is specific to the tasks used, and thus much further investigations are required before concluding that comprehension levels in the trained individual in Study 2 were the same regardless of reading speed. Actually, comprehension scores of FT were somewhat lower, though not statistically significantly, than those of the untrained participants. In addition, as described in the Introduction, the present study used relatively easy text that does not require specialized knowledge/schema on specific areas. Thus, the present results may apply only to comparable types of text and reading strategies. Other types of reading (e.g. comprehension during student study) may require more complex comprehension, such as integration of the contexts with prior knowledge or understanding of complex concepts. It would surely be required to further modify comprehension questions by using more various types of text, so that different types of comprehension could be separately examined. In addition, there is so far no clear evidence that any participants including the trainees could comprehend the details of non-fixated text. This also needs to be examined using modified procedures.

Fifth, even though the instructed reading strategies changed across Studies 1 and 2, little difference in the reading speed, comprehension, or eye-movement measures was found for the untrained participants. It is thus possible that the change in instructions had little impact on the reading strategies. However, this point remains unclear for the trainees in Studies 1 and 2, whose levels of expertise were different. It would thus be necessary to further examine how trained participants may perform under different instructions.

The fact that FT appeared to understand the text without gazing over the whole sentence areas suggests the possibility that effective perceptual span may have been widened through the course of training. This seems consistent with the preliminary finding that performance on visual search tasks including conjunction search tasks was better in the trainees on this method than in the untrained participants [40]. Findings from fMRI studies suggesting enhanced visuospatial processing in speed-readers [19] seem consistent with the notion as well. It is worth noting that FT’s pattern of fixations as shown in **Figures 4**(**A**) and **5**(**B**) suggest an asymmetric perceptual span in the downward direction, because such patterns of the perceptual span do not seem to have been demonstrated in languages read from top to bottom. At the neuronal level, it seems possible that trained readers may exhibit different activation patterns for the same tasks, thus possibly using neural networks differently than untrained participants. In addition to the use of fewer phonological processes compared with untrained readers, speed-readers may use different neural pathways while reading Japanese sentences. Typically, in reading Japanese sentences two different neural pathways for processing Japanese *kanji* (morphograms) and *kana* (phonogram) characters are used [41]–[42]. However, preliminary findings suggested that trained speed-readers might use fewer neural processes for reading *kana* than untrained participants [43]. Much further behavioral and neuroimaging studies will be necessary to elucidate such differences in relation to the neural pathways and effective perceptual span.

From a methodological perspective, the present research made use of Tobii eye-tracking system in a reading situation. The method can be safely used not only for adults but also for children [28], [44] in naturalistic settings. It may thus potentially enable further related investigations, including reading development or evaluation of disorders of reading [45]–[47] and reading in foreign languages [48]. Because no devices have to be attached to the participants’ bodies, it also enables simultaneous recordings with other non-invasive neuroimaging techniques such as near-infrared spectroscopy (NIRS) [49]–[51] and electroencephalogram (EEG) [52]. However, a number of weak points of the present method also justify the notion that use of eye-tracking methods having much higher temporal and spatial resolution would be desired for the future quest. First, it should be noted that a 60 Hz sampling rate of the eye-tracker used in this study was relatively low for reading studies, even though other reading studies used this rate [26]. Because this may have reduced sensitivity to small differences in fixation durations, such as the first-pass reading measures, a higher recording rate would be desired in the future quest. Second, even though defining a fixation as stability within 0.9 degrees in visual angle as done in this study seems a standard practice for the present eye-tracker, this definition may have hidden refixations that would be detected as separate fixations with more accurate equipment. Also, it appears better if more detailed local analyses, such as reading behavior for specific critical words, could be effectively used, which should provide more fine grained analyses. Use of more accurate eye-tracking methods than the present one seems to be required in this respect as well.

To summarize, in addition to finding correlations between reading speed and comprehension scores and eye-movement measures in reading Japanese contemporary novels by normal untrained readers, we also obtained cases by trainees of a speed-reading method regarding relationships between these variables. The trainees overall showed poor performance influenced by the speed-accuracy trade-off, although this trade-off appeared to be reduced in the case of one high-level expert, who used horizontal eye-movements. Effective use of eye-tracking systems in computer-assisted settings would allow for potential applications in reading studies as well as in studies of speed-reading. By accumulating both behavioral and neurocognitive evidence by using various experimental tasks and modified procedures, it would be possible to further elucidate the potentials of humans’ cognitive capabilities that may be achieved through meditation-based visual training.
